# Supplementary material for: Social stratification reflected in bone mineral density and stature: Spectral imaging and osteoarchaeological findings from medieval Norway
Source: PLoS One. 2022 Oct 19;17(10):e0275448. doi: 10.1371/journal.pone.0275448 (PMC9581373; doi:10.1371/journal.pone.0275448)
Supplement: S1 Table — (PDF) [file pone.0275448.s001.pdf]

Supporting information S1 file

S1 Table. Data on age, sex, SES, BMD and stature for the total study sample.

Skeletal material reposited in the Schreiner Collection, University of Oslo, Norway.

Burial site	County	Ant. no*	Mean BMD	Sex**	Age***	SES****	Stature
Church of Prestgard	Innlandet	3533	1.350	1	MA	2	169
Church of Prestgard	Innlandet	3534	0.800	2	OA	2	173
Church of Prestgard	Innlandet	3535	1.239	1	OA	2	171
Church of Prestgard	Innlandet	3536	0.794	2	OA	2	171
Church of Prestgard	Innlandet	3538	0.912	2	OA	2	167
Church of Prestgard	Innlandet	3539	0.938	1	OA	2	163
Church of Prestgard	Innlandet	3540	0.845	2	OA	2	170
Church of Prestgard	Innlandet	3541	0.549	1	OA	2	158
Church of Prestgard	Innlandet	3542	1.135	1	MA	2	163
Church of Prestgard	Innlandet	3543	0.895	2	OA	2	172
Church of Prestgard	Innlandet	3544	1.103	1	OA	2	173
Church of Prestgard	Innlandet	3546	0.895	1	YA	2	159
Church of Prestgard	Innlandet	3547	0.953	2	OA	2	185
Church of Prestgard	Innlandet	3549	1.005	2	MA	2	175
Church of Prestgard	Innlandet	3550	0.852	2	OA	2	176
Church of Prestgard	Innlandet	3551	0.659	1	MA	2	162
Church of Prestgard	Innlandet	3552	0.981	2	MA	2	166
Church of Prestgard	Innlandet	3554	0.818	2	OA	2	170
Church of Prestgard	Innlandet	3555	0.748	2	YA	2	174
Church of Prestgard	Innlandet	3556	1.026	2	MA	2	173
Church of Prestgard	Innlandet	3557	1.062	1	YA	2	160
Church of Prestgard	Innlandet	3558	0.852	2	OA	2	182
Church of Prestgard	Innlandet	3559	0.949	1	OA	2	164
Church of Prestgard	Innlandet	3561	0.844	1	MA	2	154
Church of Prestgard	Innlandet	3562	0.958	1	OA	2	170
Church of Prestgard	Innlandet	3563	1.396	2	MA	2	172
Church of Prestgard	Innlandet	3564	1.026	2	YA	2	165
Church of Prestgard	Innlandet	3565	0.889	1	OA	2	164
Church of Prestgard	Innlandet	3567	0.796	1	MA	2	157
Church of Prestgard	Innlandet	3568	0.739	1	OA	2	159
Church of Prestgard	Innlandet	3609	0.756	2	OA	2	175
Church of St. Clemens	Oslo	1943	1.029	2	OA	2	174
Church of St. Clemens	Oslo	2562	1.304	2	YA	2	171
Church of St. Clemens	Oslo	2564	1.177	2	YA	2	161
Church of St. Clemens	Oslo	2565	0.865	1	OA	2	158
Church of St. Clemens	Oslo	2566	0.881	1	YA	2	157
Church of St. Clemens	Oslo	2567	0.770	1	OA	2	157
Church of St. Clemens	Oslo	2568	1.010	1	YA	2	159
Church of St. Clemens	Oslo	2571	1.160	1	OA	2	160
Church of St. Clemens	Oslo	2573	1.016	1	MA	2	162
Church of St. Clemens	Oslo	2574	0.989	2	MA	2	170
Church of St. Clemens	Oslo	2575	1.420	1	YA	2	169
Church of St. Clemens	Oslo	2577	0.646	1	MA	2	165
Church of St. Clemens	Oslo	2578	1.063	2	OA	2	174
Church of St. Clemens	Oslo	2579	0.731	1	OA	2	166
Church of St. Clemens	Oslo	2580	1.205	2	MA	2	180
Church of St. Clemens	Oslo	2586	1.037	2	YA	2	173
Church of St. Clemens	Oslo	2587	1.207	2	YA	2	175
Church of St. Clemens	Oslo	2588	1.008	2	YA	2	174
Church of St. Clemens	Oslo	2590	0.989	1	YA	2	155
Church of St. Mary	Oslo	4186	0.911	2	MA	1	171
Church of St. Mary	Oslo	4187	0.924	2	YA	1	178
Church of St. Mary	Oslo	4188	1.085	1	OA	1	170
Church of St. Mary	Oslo	4189	1.104	2	OA	1	175
Church of St. Mary	Oslo	4190	0.877	1	MA	1	167
Church of St. Mary	Oslo	4191	1.110	1	YA	1	173
Church of St. Mary	Oslo	4193	1.083	2	MA	1	178
Church of St. Mary	Oslo	4195	0.964	2	OA	1	178
Church of St. Mary	Oslo	4196	0.957	2	OA	1	179
Church of St. Mary	Oslo	4197	0.929	2	MA	1	173
Church of St. Mary	Oslo	4198	1.090	1	MA	1	168
Church of St. Mary	Oslo	4199	0.897	1	MA	1	151
Church of St. Mary	Oslo	4203	0.792	2	OA	1	182
Church of St. Mary	Oslo	4204	1.105	2	MA	1	185
Church of St. Mary	Oslo	4206	1.023	1	YA	1	171
Church of St. Mary	Oslo	4207	1.128	2	YA	1	164
Church of St. Mary	Oslo	4208	1.177	2	MA	1	171
Church of St. Mary	Oslo	4210	1.135	2	MA	1	165
Church of St. Mary	Oslo	4788	0.958	1	YA	1	159
Church of St. Mary	Oslo	4794	0.997	2	OA	1	169
Church of St. Mary	Oslo	4795	1.337	2	OA	1	169
Church of St. Mary	Oslo	4796	0.961	2	OA	1	174
Church of St. Mary	Oslo	4797	1.130	1	MA	1	163
Church of St. Mary	Oslo	4802	0.917	2	OA	1	172
Church of St. Mary	Oslo	4807	1.088	2	YA	1	169
Church of St. Mary	Oslo	4885	1.170	2	MA	1	186
Church of St. Mary	Oslo	4887	1.095	2	MA	1	175
Church of St. Mary	Oslo	4888	1.224	1	YA	1	170
Church of St. Mary	Oslo	4889	1.332	2	YA	1	179

Church of St. Mary	Oslo	4890	1.029	2	YA	1	178
Church of St. Mary	Oslo	4892	0.933	1	OA	1	168
Church of St. Mary	Oslo	4898	1.030	1	YA	1	166
Church of St. Mary	Oslo	4902	1.048	2	MA	1	175
Church of St. Mary	Oslo	4943	0.788	2	OA	1	181
Church of St. Mary	Oslo	4947	0.856	1	MA	1	168
Church of St. Mary	Oslo	4948	1.165	2	YA	1	184
Church of St. Mary	Oslo	4949	0.998	2	OA	1	178
Church of St. Mary	Oslo	4950	1.198	2	OA	1	168
Church of St. Mary	Oslo	4951	0.924	2	OA	1	179
Church of St. Mary	Oslo	4955	0.718	1	MA	1	156
Church of St. Mary	Oslo	4960	1.015	2	MA	1	170
Church of St. Mary	Oslo	4961	1.109	2	YA	1	180
Church of St. Mary	Oslo	4962	1.179	2	YA	1	175
Church of St. Mary	Oslo	4968	0.998	1	YA	1	154
Church of St. Mary	Oslo	4972	0.714	1	MA	1	159
Church of St. Mary	Oslo	4973	1.358	2	YA	1	177
Church of St. Mary	Oslo	4974	1.408	2	YA	1	178
Church of St. Mary	Oslo	4979	1.201	2	MA	1	169
Church of St. Mary	Oslo	4981	0.850	2	MA	1	176
Church of St. Mary	Oslo	4983	1.016	2	YA	1	179
Church of St. Mary	Oslo	5134	1.012	2	MA	1	178
Church of St. Mary	Oslo	4783	1.202	1	YA	1	171
Church of St. Mary	Oslo	4784	0.938	2	YA	1	175
Church of St. Mary	Oslo	4785	0.859	2	MA	1	180
Hamar Cathedral	Innlandet	7472	1.043	2	OA	2	183
Hamar Cathedral	Innlandet	7481	0.978	2	MA	2	174
Hamar Cathedral	Innlandet	7491	1.070	1	YA	2	162
Hamar Cathedral	Innlandet	7493	0.921	2	YA	2	173
Hamar Cathedral	Innlandet	7494	0.852	2	MA	2	174
Hamar Cathedral	Innlandet	7499	1.364	2	YA	2	173
Hamar Cathedral	Innlandet	7501	1.270	2	MA	2	179
Hamar Cathedral	Innlandet	7502	1.168	2	YA	2	177
Hamar Cathedral	Innlandet	7511	1.266	2	YA	2	174
Hamar Cathedral	Innlandet	7521	0.946	2	MA	2	176
Hamar Cathedral	Innlandet	7535	1.098	1	MA	2	166
Hamar Cathedral	Innlandet	7548	0.942	1	OA	2	162
Hamar Cathedral	Innlandet	7579	0.744	1	OA	2	155
Hamar Cathedral	Innlandet	7610	1.065	2	OA	2	166
Hamar Cathedral	Innlandet	7613	0.938	1	OA	2	162
Hamar Cathedral	Innlandet	7616	0.798	2	OA	2	174
Hamar Cathedral	Innlandet	7619	0.937	1	YA	2	155
Hamar Cathedral	Innlandet	7624	1.196	1	MA	2	158
Hamar Cathedral	Innlandet	7651	1.167	1	MA	2	157
Hamar Cathedral	Innlandet	7661	0.754	1	OA	2	156
Hamar Cathedral	Innlandet	7664	0.859	1	OA	2	163
Hamar Cathedral	Innlandet	7665	0.799	1	OA	2	163
Hamar Cathedral	Innlandet	7682	0.977	1	MA	2	161
Hamar Cathedral	Innlandet	7692	0.817	1	OA	2	164
Hamar Cathedral	Innlandet	7695	0.706	1	OA	2	162
Hamar Cathedral	Innlandet	7699	0.853	1	OA	2	157
Hamar Cathedral	Innlandet	7711	0.676	1	OA	2	157
Hamar Cathedral	Innlandet	7735	0.702	1	MA	2	160
Hamar Cathedral	Innlandet	7741	0.712	1	MA	2	155
Hamar Cathedral	Innlandet	7775	0.709	1	OA	2	152
Hamar Cathedral	Innlandet	7806	0.728	1	OA	2	153
Hamar Cathedral	Innlandet	7811	0.820	2	OA	2	166
Hamar Cathedral	Innlandet	7876	0.981	1	MA	2	163
Hamar Cathedral	Innlandet	7881	1.010	2	YA	2	167
Hamar Cathedral	Innlandet	7889	1.090	2	YA	2	170
Hamar Cathedral	Innlandet	7912	1.274	2	YA	2	189
Hamar Cathedral	Innlandet	7916	1.177	2	OA	2	181
Hamar Cathedral	Innlandet	7482	0.942	1	OA	2	163
Hamar Cathedral	Innlandet	7486	0.614	1	OA	2	153
Hamar Cathedral	Innlandet	7500	1.156	2	YA	2	190
Hamar Cathedral	Innlandet	7507	0.988	1	YA	2	161
Hamar Cathedral	Innlandet	7517	0.904	1	YA	2	160
Hamar Cathedral	Innlandet	7524	1.079	2	OA	2	183
Hamar Cathedral	Innlandet	7527	1.016	1	OA	2	151
Hamar Cathedral	Innlandet	7530	0.674	1	OA	2	158
Hamar Cathedral	Innlandet	7534	0.681	1	OA	2	159
Hamar Cathedral	Innlandet	7536	1.199	1	MA	2	155
Hamar Cathedral	Innlandet	7544	0.998	2	MA	2	168
Hamar Cathedral	Innlandet	7549	0.944	2	MA	2	173
Hamar Cathedral	Innlandet	7552	1.058	1	YA	2	160
Hamar Cathedral	Innlandet	7563	1.371	2	OA	2	180
Hamar Cathedral	Innlandet	7575	0.929	2	MA	2	175
Hamar Cathedral	Innlandet	7577	0.846	2	YA	2	178
Hamar Cathedral	Innlandet	7588	1.239	2	YA	2	179
Hamar Cathedral	Innlandet	7590	1.013	2	OA	2	164
Hamar Cathedral	Innlandet	7599	0.656	1	MA	2	157
Hamar Cathedral	Innlandet	7603	0.877	1	OA	2	168
Hamar Cathedral	Innlandet	7608	0.849	1	OA	2	169
Hamar Cathedral	Innlandet	7611	0.900	2	MA	2	167
Hamar Cathedral	Innlandet	7612	0.752	1	OA	2	155
Hamar Cathedral	Innlandet	7617	0.832	2	MA	2	180
Hamar Cathedral	Innlandet	7622	0.574	1	OA	2	157

Hamar Cathedral	Innlandet	7623	1.041	2	MA	2	175
Hamar Cathedral	Innlandet	7629	1.050	2	MA	2	170
Hamar Cathedral	Innlandet	7630	1.064	2	YA	2	177
Hamar Cathedral	Innlandet	7632	0.601	1	MA	2	161
Hamar Cathedral	Innlandet	7633	1.080	2	YA	2	173
Hamar Cathedral	Innlandet	7634	0.776	1	MA	2	154
Hamar Cathedral	Innlandet	7635	0.926	2	MA	2	177
Hamar Cathedral	Innlandet	7638	1.159	2	MA	2	179
Hamar Cathedral	Innlandet	7656	0.711	1	OA	2	160
Hamar Cathedral	Innlandet	7659	1.006	1	MA	2	159
Hamar Cathedral	Innlandet	7662	0.899	2	YA	2	178
Hamar Cathedral	Innlandet	7667	0.687	1	OA	2	166
Hamar Cathedral	Innlandet	7668	1.241	2	MA	2	177
Hamar Cathedral	Innlandet	7673	0.730	1	YA	2	161
Hamar Cathedral	Innlandet	7687	0.793	1	OA	2	168
Hamar Cathedral	Innlandet	7707	1.003	2	YA	2	170
Hamar Cathedral	Innlandet	7716	1.045	2	YA	2	181
Hamar Cathedral	Innlandet	7719	1.022	2	OA	2	182
Hamar Cathedral	Innlandet	7722	0.845	1	OA	2	170
Hamar Cathedral	Innlandet	7725	1.076	1	YA	2	167
Hamar Cathedral	Innlandet	7733	1.022	2	YA	2	180
Hamar Cathedral	Innlandet	7736	0.771	1	MA	2	163
Hamar Cathedral	Innlandet	7744	1.178	1	YA	2	163
Hamar Cathedral	Innlandet	7751	0.853	1	OA	2	158
Hamar Cathedral	Innlandet	7756	1.010	2	YA	2	167
Hamar Cathedral	Innlandet	7767	0.602	1	OA	2	158
Hamar Cathedral	Innlandet	7769	0.983	2	YA	2	176
Hamar Cathedral	Innlandet	7773	1.283	2	YA	2	186
Hamar Cathedral	Innlandet	7778	1.121	2	YA	2	179
Hamar Cathedral	Innlandet	7779	0.878	2	YA	2	173
Hamar Cathedral	Innlandet	7783	0.773	1	MA	2	158
Hamar Cathedral	Innlandet	7786	0.792	1	MA	2	156
Hamar Cathedral	Innlandet	7790	1.031	2	YA	2	171
Hamar Cathedral	Innlandet	7802	0.730	1	OA	2	166
Hamar Cathedral	Innlandet	7842	0.774	2	OA	2	177
Hamar Cathedral	Innlandet	7849	0.774	2	OA	2	172
Hamar Cathedral	Innlandet	7878	1.044	2	YA	2	168
Hamar Cathedral	Innlandet	7900	0.938	2	OA	2	182
Hamar Cathedral	Innlandet	7922	1.098	2	YA	2	172
Hamar Cathedral	Innlandet	7932	1.146	2	YA	2	178
St. Olav's Monastery	Oslo	3495	1.207	2	MA	1	176
St. Olav's Monastery	Oslo	3496	1.074	1	YA	1	165
St. Olav's Monastery	Oslo	3499	0.929	1	OA	1	164
St. Olav's Monastery	Oslo	3501	1.350	2	YA	1	169
St. Olav's Monastery	Oslo	3502	0.954	1	OA	1	164
St. Olav's Monastery	Oslo	3503	0.974	2	OA	1	172
St. Olav's Monastery	Oslo	3504	1.218	1	YA	1	161
St. Olav's Monastery	Oslo	3505	1.136	1	YA	1	170
St. Olav's Monastery	Oslo	3506	1.208	2	MA	1	178
St. Olav's Monastery	Oslo	3507	1.040	1	YA	1	166
St. Olav's Monastery	Oslo	3508	0.982	1	MA	1	161
St. Olav's Monastery	Oslo	3509	0.820	1	MA	1	155
St. Olav's Monastery	Oslo	3510	1.165	2	OA	1	182
St. Olav's Monastery	Oslo	3511	0.905	2	MA	1	172
St. Olav's Monastery	Oslo	3513	1.252	2	YA	1	172
St. Olav's Monastery	Oslo	3514	0.902	2	OA	1	180
St. Olav's Monastery	Oslo	3515	1.016	2	YA	1	176
St. Olav's Monastery	Oslo	3516	1.674	2	YA	1	178
St. Olav's Monastery	Oslo	3774	1.176	2	YA	1	169
St. Olav's Monastery	Oslo	3775	0.922	2	MA	1	175
St. Olav's Monastery	Oslo	3776	1.110	1	YA	1	165

* Ant.no= Anthropological number in the Schreiner Collection, University of Oslo

** Sex 1= female, Sex 2= male

*** YA (Young Adult)= mean 27 years, MA (Middle Adult)= mean 42,5 years, OA (Old Adult)= mean 50 years.

**** SES group 1 = high status, SES group 2= parish population.
